# Emulsified Nanoparticles Containing Inactivated Influenza Virus and CpG Oligodeoxynucleotides Critically Influences the Host Immune Responses in Mice

**DOI:** 10.1371/journal.pone.0012279

**Published:** 2010-08-19

**Authors:** Ming-Hsi Huang, Su-Chen Lin, Chia-Hsin Hsiao, Hsin-Ju Chao, Hung-Ren Yang, Chien-Chun Liao, Po-Wei Chuang, Huang-Pi Wu, Chiung-Yi Huang, Chih-Hsiang Leng, Shih-Jen Liu, Hsin-Wei Chen, Ai-Hsiang Chou, Alan Yung-Chih Hu, Pele Chong

**Affiliations:** Vaccine Research and Development Center, National Health Research Institutes, Zhunan Town, Taiwan; Veterinary Laboratories Agency, United Kingdom

## Abstract

**Background:**

Antigen sparing and cross-protective immunity are regarded as crucial in pandemic influenza vaccine development. Both targets can be achieved by adjuvantation strategy to elicit a robust and broadened immune response. We assessed the immunogenicity of an inactivated H5N1 whole-virion vaccine (A/Vietnam/1194/2004 NIBRG-14, clade 1) formulated with emulsified nanoparticles and investigated whether it can induce cross-clade protecting immunity.

**Methodology/Principal Findings:**

After formulation with PELC, a proprietary water-in-oil-in-water nanoemulsion comprising of bioresorbable polymer/Span®85/squalene, inactivated virus was intramuscularly administered to mice in either one-dose or two-dose schedule. We found that the antigen-specific serum antibody responses elicited after two doses of non-adjuvanted vaccine were lower than those observed after a single dose of adjuvanted vaccine, PELC and the conventional alum adjuvant as well. Moreover, 5 µg HA of PELC-formulated inactivated virus were capable of inducing higher antibodies than those obtained from alum-adjuvanted vaccine. In single-dose study, we found that encapsulating inactivated virus into emulsified PELC nanoparticles could induce better antibody responses than those formulated with PELC-adsorbed vaccine. However, the potency was rather reduced when the inactivated virus and CpG (an immunostimulatory oligodeoxynucleotide containing unmethylated cytosine-guanosine motifs) were co-encapsulated within the emulsion. Finally, the mice who received PELC/CpG(adsorption)-vaccine could easily and quickly reach 100% of seroprotection against a homologous virus strain and effective cross-protection against a heterologous virus strain (A/Whooper swan/Mongolia/244/2005, clade 2.2).

**Conclusions/Significance:**

Encapsulating inactivated H5N1 influenza virus and CpG into emulsified nanoparticles critically influences the humoral responses against pandemic influenza. These results demonstrated that the use of PELC could be as antigen-sparing in preparation for a potential shortage of prophylactic vaccines against local infectious diseases, in particular pandemic influenza. Moreover, the cross-clade neutralizing antibody responses data verify the potential of such adjuvanted H5N1 candidate vaccine as an effective tool in pre-pandemic preparedness.

## Introduction

Vaccination is the best cost-effective biomedical approach in the face of the threat of the emerging diseases like influenza epidemics and pandemics [1], [2]. In preparedness of influenza pandemic vaccine, two of the key challenges are to produce sufficient quantities of vaccine in a narrowed time window and to induce significant immunogenicity and cross-protective immunity after vaccine injections [1]–[3]. Fortunately, both targets can be achieved by using an additive substance dubbed adjuvant to elicit a robust and broadened immune response [3].

Despite the excitement about how adjuvants work, alum (a term for aluminum-based mineral salts) is the only adjuvant approved by the U.S. Food and Drug Administration (FDA) in the influenza vaccines [3]. However, highly heterogeneous, difficult to manufacture in a consistent and reproducible manner, and a boost injection required to generate protection are obstacles which limited alum in influenza vaccine use [4], [5]. In fact, it is also hypothesized that certain antigens do not adsorb well onto alum due to the presence of the same charge on the adjuvant and antigens. In order to reach high adsorption capacity, alum requires preparation in a slightly acidic environment (pH = 6) [6].

Among the vaccine adjuvants evaluated in human trials, oligodeoxynucleotides (ODNs) containing unmethylated cytosine-guanosine motifs (CpG) are well-known inducers of the innate immune response through activation of toll-like receptor (TLR)-9, which is known an intracellular receptor within the endosomal compartments of immune cells [7]. It has also been shown to induce T help 1 (Th1) immune responses, characterized by secretion of interferon (IFN)-γ and the generation of IgG2a immunoglobulin subclass in mouse model [8], [9]. Although CpG was proved as an adjuvant for a wide range of antigens [7], [8], it was also observed that CpG alone did not appear to be a potent adjuvant in some cases like HIV and influenza antigens [9], [10]. To this end, a number of studies have shown that immune responses could be improved by delivering CpG directly to the immune cells [10], [11].

In preparation for a potential shortage of pandemic influenza vaccine, we have previously developed the production process for the World Health Organization (WHO) vaccine strain NIBRG-14 (recombinant clade 1 H5N1 isolate A/Vietnam/1194/2004 engineered by reverse genetics) using Madin-Darby canine kidney (MDCK) cells, growing either in roller-bottles (*Chong et al. Emergency production of avian flu vaccines. In: Options for the control of influenza VI. Toronto, Canada, 2007: P291.*) or microcarrier-based cell culture system [2]. We found that inactivated virus adjuvanted with alum could elicit high virus neutralizing antibody titers in different animal models after 2 to 3 immunizations, and also conferred protection in mice against the wild type H5N1 challenges. Regarding the diversity, H5N1 influenza viruses can be broadly divisible into ten distinct lineages or clades, and multiple subclades within clade 2. The vaccine candidate NIBRG-14 belongs to clade 1 and the strains that have continued to spread in Southeast Asia are clade 2 [1]. For the feasibility study on a pre-pandemic vaccination, it will be important to evaluate whether the use of adjuvants can increase the cross-protective immune responses against other clades of H5N1 viruses.

Recently, based on a bioresorbable diblock tri-component copolymer poly(ethylene glycol)-block-poly(lactide-co-ε-caprolactone) (PEG-*b*-PLACL), we developed and optimized a water-in-oil-in-water (W/O/W) multiphase emulsion-type vaccine delivery system called PELC [12], [13]. Preliminary immunogenicity studies showed that following a single injection in mice, the PELC-formulated 0.5 µg hemagglutinin (HA) of inactivated H5N1 virus induced more potent antigen-specific antibodies titers than 5 µg HA of non-adjuvanted virus. In addition, T-cell proliferative responses and IFN-γ cytokine secretion were significantly increased when the inactivated virus was formulated with PELC [13]. Moreover, the vaccine formulation with PELC did not skew the immune response toward Th1 or Th2. These results indicate that PELC can be used for effective single-dose immunization and thus play an important role in antigen-sparing influenza pandemic preparedness. Even though it is desirable that vaccines for pandemic pathogens would provide protective immunity after a single administration, one cannot conclude from mouse data that a single injection with PELC-formulated H5N1 vaccine would be sufficient in humans. Therefore, it would have been interesting to see what the responses in mice are after a boost injection.

In this study, we aimed to deepen understanding the role of PELC in vaccine immunogenicity. First, we evaluated the boosting immune response of mice that already received a single injection of either non-adjuvanted or PELC-adjuvanted NIBRG-14 vaccine. We also planed to study on PELC for its ability to adjuvant NIBRG-14 vaccine in different strategies, adsorption or encapsulation. Subsequently, the improvement of the potency of PELC by combination of immunostimulatory CpG ODNs to manipulate the titers of protective antibodies against the homologous virus strain and a heterologous Mongolia/244 prototype virus strain derived by reverse genetics from a drifted H5N1 isolate (A/Whooper swan/Mongolia/244/2005, clade 2.2) was also investigated. The immunogenicity of a H5N1 vaccine candidate after formulated with PELC and/or CpG was determined in mice for induction of humoral responses following prime/boost or single-dose immunization schedules. The results were compared with those obtained from conventional alum adjuvant.

## Results

### Formulating PELC with inactivated influenza virus and CpG

**Figure 1:**
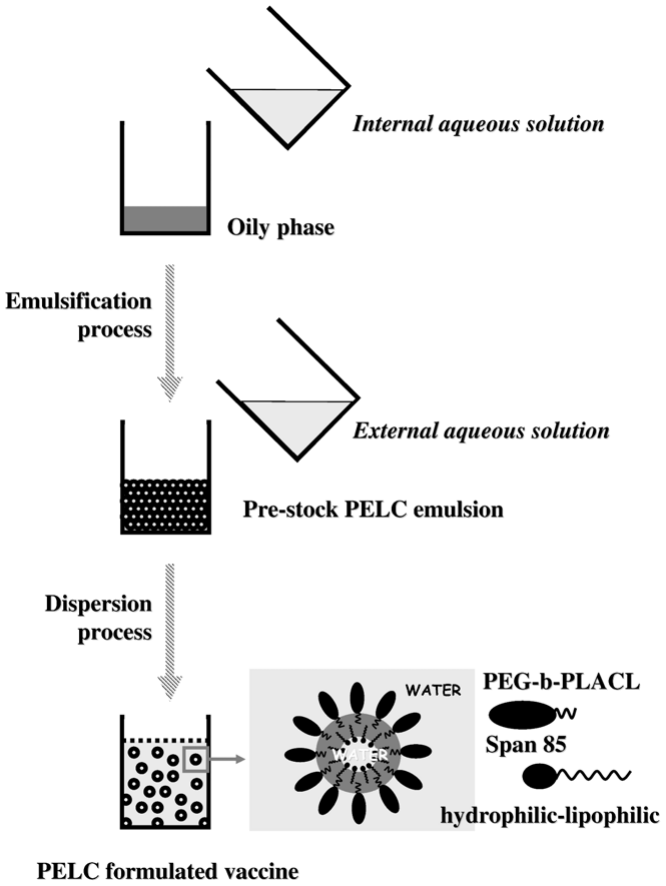
Schematic presentation of the PELC-formulated vaccine preparation. Typically, PELC-adjuvanted vaccine follows a two-step procedure including emulsification and dispersion. In the first step, aqueous solution containing PEG-*b*-PLACL and oily phase consisting squalene and Span®85 were homogenized to form a stable and isotropic emulsion, PELC. In this case, oil droplets (stabilized by hydrophilic PEG-*b*-PLACL) dispersed into the continuous water, and the core oil (stabilized by lipophilic Span®85) also entrapped aqueous. The polymer-stabilized emulsion has high affinity to water, so that the pre-emulsified stock could be further dispersed into aqueous solution to form homogeneous nanoparticles in the second step.

**Figure 1** shows the preparation of the PELC-formulated vaccine. Initially, phase separation occurred between aqueous solution and water-immiscible oily phase, where the aqueous solution comprises phosphate buffered saline (PBS) and PEG-*b*-PLACL, and the oily phase comprises squalene and Span®85. Following the emulsification, a stable and isotropic emulsion dubbed PELC was obtained. Physiochemical studies using droplet test and *in vitro* release have implied the polymer-stabilized PELC emulsion has surface with high affinity to water; furthermore, the squalene core (stabilized by lipophilic Span®85) of PELC also entrapped aqueous [12]. Due to the emulsion stock is too viscous to be injected by syringe, a further dispersion step into the majority of aqueous was performed to increase the fluidity, thus yielding stable and injectable W/O/W nanoemulsion, i.e. the core oil entrapped aqueous solution (*internal aqueous solution*), and the oil droplets also dispersed into the continuous solution (*external aqueous solution*). Antigen or water-soluble bioactive substance may be dissolved either in the internal and/or external aqueous solution of the W/O/W emulsion. For a controlled release formulation, dissolving and encapsulating an antigen in the internal aqueous phase has the effect of protecting the antigen. The antigens (or biological active substances) trapped within the polymer-emulsified oily emulsion will be released mostly by diffusion from the core oil to the surface, but also to a lesser extent by degradation mechanisms and emulsion breaks [12], [14]. Conversely, dissolving and entrapping an antigen in the external aqueous phase has the effect of facilitating the expression of the antigen.

We thus defined the PELC-formulated vaccines as PELC(encapsulation) and PELC/CpG(encapsulation) when the antigen of inactivated influenza virus and/or CpG were introduced in the *internal aqueous solution*. Meanwhile, PELC(adsorption) and PELC/CpG(adsorption) were represented the vaccine formulations as the inactivated influenza virus and/or CpG were introduced in the *external aqueous solution*. The vaccine design, manufacture process, and particle size of the four emulsified vaccines were summarized in **Table 1**. Homogeneous particles with mean size ranged from 400 to 500 nm were observed. Virus antigen and/or CpG are not a key point to the particle size of the emulsions, probably due to small amount of antigen or CpG content in the vaccine formulations. In fact, CpG is well dissolved in the aqueous solution, while the size of H5N1 virus suspension is about 100 nm [2].

**Table 1:** Vaccine design, manufacture process, and particle size of four emulsions.

Formulation	Emulsification process^a^		Dispersion process^b^	Particle size^c^ (Mean ± STD, nm)
	Internal aqueous solution	Oily phase	External aqueous solution	
PELC(encapsulation)	PBS/PEG-b-PLACL/virus	Squalene/Span®85	PBS	470±20
PELC/CpG(encapsulation)	PBS/PEG-b-PLACL/virus/CpG	Squalene/Span®85	PBS	380±40
PELC(adsorption)	PBS/PEG-b-PLACL	Squalene/Span®85	PBS/virus	390±70
PELC/CpG(adsorption)	PBS/PEG-b-PLACL	Squalene/Span®85	PBS/virus/CpG	400±30

a Emulsification process was performed using homogeniser under 6,000 rpm for 5 min.

b Dispersion process was performed using test-tube rotator under 5 rpm at least 1 hr.

c The data were represented as the mean with standard deviation (STD) of three samples.

### A single-dose nanoemulsion-formulated pandemic influenza vaccine induces more potent antigen-specific serum antibody responses than a two-dose non-adjuvanted vaccine

**Figure 2:**
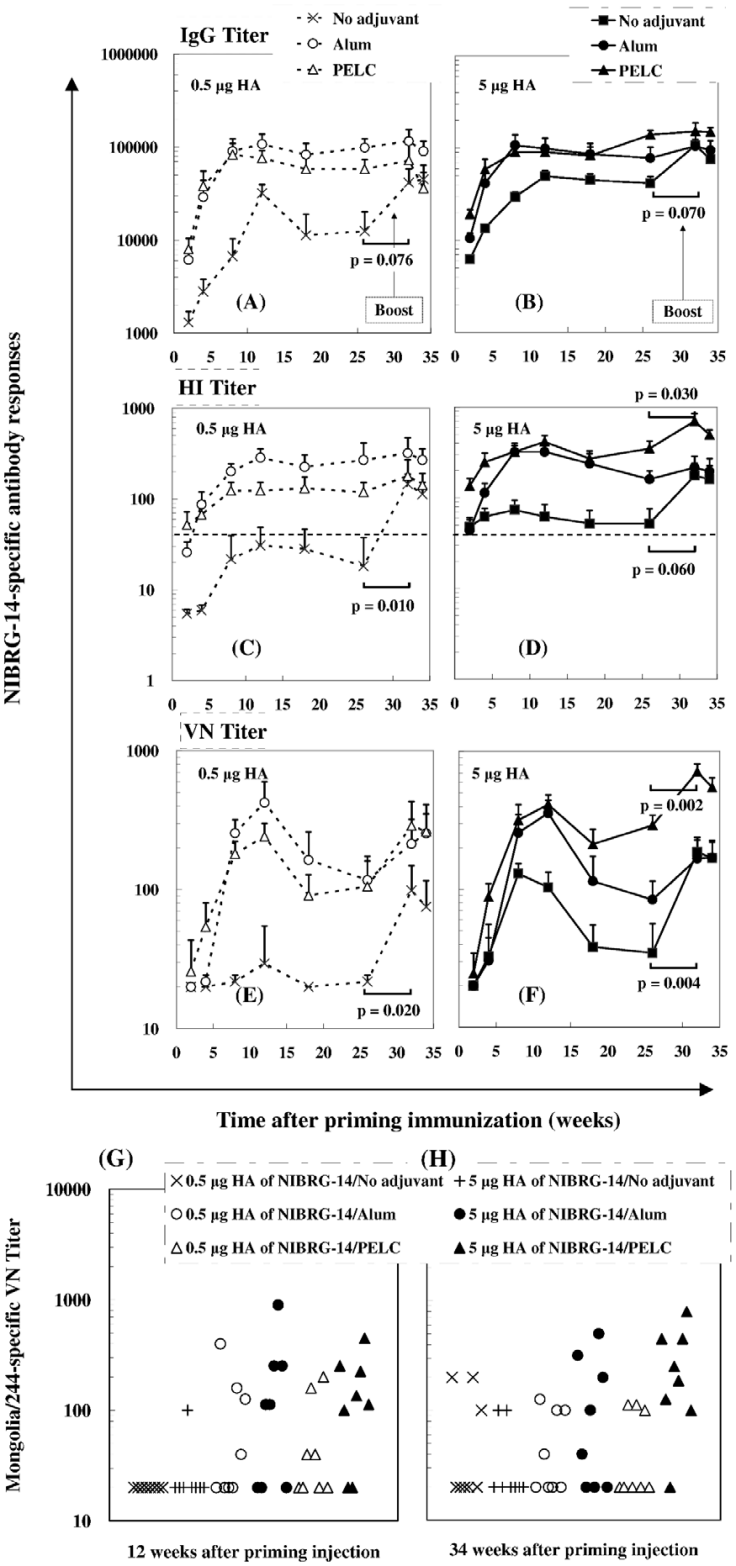
NIBRG-14-specific (A, B) IgG, (C, D) HI, (E, F) VN antibody responses and (G, H) Mongolia/244-specific VN antibodies in BALB/c mice immunized by different prime/boost vaccination schedules. Mice were primed i.m. with 0.5 µg or 5 µg HA of inactivated NIBRG-14 virus, either with antigen in PBS or adsorbed with alum or PELC. At week 30, all mice were boosted i.m. with 0.5 µg HA non-adjuvanted inactivated NIBRG-14 virus vaccine. Serum samples were collected from immunized mice and the antibody titers were determined by ELISA, HI and VN immunoassays. The data are presented as geometric mean titers (GMT) with standard errors (SE) of eight mice per group. *p*-value: Comparison between the boosting dose. The dotted horizontal line represents an HI titer of 40.

To evaluate whether the antigen-specific antibodies can be drove by a boost immunization, BALB/c mice were immunized with 0.5 µg or 5 µg HA inactivated NIBRG-14 virus, either with antigen in PBS or adsorbed with alum or PELC. At week 30, all mice were boosted with dose of 0.5 µg HA H5N1 non-adjuvanted inactivated virus vaccine. The elicited antigen-specific antibodies are shown in **Figure 2**. Following the priming injection, sera from mice vaccinated with 0.5 µg HA of non-adjuvanted inactivated virus elicited a NIBRG-14-specific antibody IgG geometric mean titer (GMT) less than 3,000 was detected at Week 2 and Week 4 (**Figure 2A**). Afterwards, it increased slowly to 6,700 at Week 8. The highest GMT response was 32,000 at Week 12, and then decreased to 11,000 at Week 18 and to 12,000 at Week 26. When the same amount of inactivated virus was co-administered with alum or PELC, the induced anti-NIBRG-14 IgG titers were significantly higher than those induced by non-adjuvanted inactivated virus. In both cases, the IgG titers peaked at Week 8 or Week 12 and fluctuated. However, there were no statistically significant differences (*p*≈1) in IgG titers between the alum- and PELC-formulated groups. After the boosting injection, the IgG titers were dramatically enhanced to 41,500 at Week 32 and 45,000 at Week 34 for group that primed non-adjuvanted inactivated virus. Nevertheless, the titers were still lower than the groups that received only one injection of alum or PELC-formulated inactivated virus vaccine. Nonetheless, the boosting effect was rather reduced in the groups of mice primed with adjuvanted vaccines compared with non-adjuvanted ones. When the amount of non-adjuvanted virus was increased to 5 µg HA, the IgG titer was enhanced during the first 8 weeks after the priming administration (**Figure 2B**), afterward, the elicited antibodies fluctuated. Similar to the responses induced from dose of 0.5 µg HA, the boosting effect was only found in the group of the mice primed with 5-µg HA non-adjuvanted vaccine.

Hemagglutination inhibition (HI) assay is the most common way to determine the efficacy of an influenza vaccine, the detected HI titer greater than 40 was read as serological protective. We determined HI activity using turkey erythrocytes incubated with sera from vaccinated groups. **Figure 2C** shows that HI antibody responses were elicited in BALB/c mice immunized with inactivated NIBRG-14 virus formulated with or without adjuvant. Overall, sera from mice vaccinated with 0.5 µg HA of non-adjuvanted inactivated virus elicited an antigen-specific HI titer less than 40 following a single injection. This finding implied that a single-dose non-adjuvanted inactivated influenza vaccine probably could not provide serological protection against the homologous virus challenge at this dosage. Alum or PELC-adjuvanted vaccines were capable of inducing higher HI titers than those developed from virus alone (P<0.05). After the second injection, the HI titers were significantly enhanced for the group the mice already received a shot of non-adjuvanted vaccine. However, it is not the case for the group the mice received a shot of alum- or PELC-adjuvanted vaccine, in those groups no boosting effect was found. When the amount of virus administrated was increased to 5 µg HA (**Figure 2D**), the mean HI titer was slightly higher than 40 in the group when the mice immunized once with inactivated virus alone, however, the seroprotection rate (mice achieving a post-vaccination titer ≥40) never reached 100%. On the other hand, after formulation with alum or PELC, seroprotection easily reached 100% in both the 0.5 µg HA and 5 µg HA groups indicating the merit of alum- or PELC-adjuvanted inactivated influenza virus. Furthermore, the boosting effect could only be found in the groups of the mice primed with non-adjuvanted or PELC-adjuvanted vaccine (*p*<0.05).

Viral neutralizing (VN) assays were also performed to provide a more functional measure of vaccine-induced immunity. As shown in **Figure 2E**, neutralizing antibodies were less than 40 from sera of mice vaccinated with 0.5 µg HA of non-adjuvanted inactivated virus. After the boost injection, the elicited antigen-specific neutralizing antibodies were detected 99 and 75 at Week 32 and Week 34, in agreement with the boosting effect found in IgG and HI titers. We also found that when the same amount of inactivated virus was co-administered with either alum or PELC, the induced anti-NIBRG-14 neutralizing titers were significantly higher than those induced by non-adjuvanted inactivated virus. 5 µg HA of PELC-formulated inactivated virus was capable of inducing higher antibodies than those obtained from alum-adjuvanted virus. Another advantage of vaccination with PELC-formulated inactivated virus was also revealed after the second administration. For non-adjuvanted vaccines, when the antigen dosage was increased to 5 µg HA, the virus neutralizing titer was only enhanced between Week 8 and Week 12 after the first administration (**Figure 2F**). After the second injection, the boosting effect was found in the groups of the mice primed non-adjuvanted or PELC-adjuvanted vaccine (*p*<0.05). In general, the antigen-specific serum antibody responses elicited after two doses of non-adjuvanted vaccine were lower than those observed after a single dose of adjuvanted vaccine, PELC and alum as well.

With respect to the induction of cross-clade neutralizing antibodies, sera from the mice that received H5N1 clade 1-NIBRG-14 vaccines were also incubated with a reassortant H5N1 clade 2-Mongolia/244 virus. Twelve weeks after priming injection, only mice vaccinated intramuscularly with adjuvanted vaccines had induced a neutralizing antibody response against the heterologous virus, alum and PELC as well (**Figure 2G**). However, high dosage (5 µg HA) was required to generate an effective protection. Finally, we also found that the second injection enhances the cross-neutralizing responses in the groups of the mice have already received 0.5 µg HA of non-adjuvanted or 5 µg HA of PELC-adjuvanted vaccine (**Figure 2H**).

### Encapsulating inactivated H5N1 influenza virus and CpG oligodeoxynucleotides into emulsified nanoparticles critically influences the humoral immune responses

In the first experiment, we found that there are no statistically significant differences in antibody responses between the groups of alum- and PELC-formulated vaccines with antigen dose of 0.5 µg HA. We next sought to improve the potency of PELC by combination of CpG ODN to manipulate the titers of protective antibodies. The elicited anti-NIBRG-14 IgG titers were shown in **Table 2**. Following a single injection, the NIBRG-14-specific antibody IgG response in the group of non-adjuvanted inactivated virus was undetectable in an initial serum dilution of 1,000 at Week 2, and less than GMT of 3,000 were detected at Week 4. Afterwards, it increased to 13,000 at Week 8, then to 16,000 at Week 12. Surprisingly, when the same amount inactivated virus was co-administered with 10 µg of CpG, the induced anti-NIBRG-14 IgG, IgG1, and IgG2a titers were less than those induced by non-adjuvanted inactivated virus. IgG titer less than GMT of 1,000 was detected within 4 weeks. Nevertheless, antibody titers were enhanced significantly when inactivated virus adsorbed onto alum or PELC. The antibody responses induced by PELC(adsorption) vaccine were slightly lower than those induced by alum-adsorbed vaccine. However, this situation could be improved when the inactivated virus were encapsulated within PELC, especially in the early stage of the post-immunization. Among the adjuvant combination systems, only PELC/CpG(adsorption) allowed to induce better antibody responses than the individual adjuvant (CpG or alum or PELC), whereas alum/CpG had this effect only in the early stage after injection. However, the potency was rather reduced when the inactivated virus and CpG were co-encapsulated within PELC. In so far as IgG subclasses were concerned, only PELC/CpG(adsorption) allows to bias the ratio of titers IgG2a/IgG1 (*p*<0.05).

**Table 2:** NIBRG-14-specific antibodies elicited in BALB/c mice following a single intramuscular dose of H5N1 inactivated virus vaccine.

Formulation	GMT ± SE (SPR, %)							
	No adjuvant	CpG	Alum	PELC (Adsorption)	PELC (Encapsulation)	Alum/CpG	PELC/CpG (Adsorption)	PELC/CpG (Encapsulation)
IgG								
Week 2	<1,000	<1,000	2,250±700	1,800±1,500	5,650±2,100*	4,500±1,200*	10,000±1,700* #	2,800±1,300*
Week 4	2,800±450	<1,000	18,000±8,700*	5,650±6,000	20,000±8,800*	40,000±22,000*	80,600±17,000* #	16,000±19,500*
Week 8	13,000±1,700	3,000±500	64,000±20,000*	14,000±9,500	45,000±8,500*	64,000±18,000*	64,000±18,000*	42,000±21,000*
Week 12	16,000±3,200	6,350±1,900	73,500±19,000*	25,000±7,800	45,000±8,700*	36,000±17,500	128,000±0* #	51,000±20,000*
Subclass at Week 8								
IgG1	2,800±1,300	<1,000	18,000±8,400*	9,000±4,400*	8,000±2,200*	14,000±4,400*	6,350±3,000	9,000±4,400*
IgG2a	8,000±1,600	2,250±500	22,600±25,000	4,500±10,000	20,000±12,000	28,000±28,000	90,500±17,000*	18,000±11,500
IgG2a/IgG1	2.8±1.3	-	1.3±1.6	0.5±0.8	2.5±3.0	2.0±3.0	14.0±9.0* #	2.0±3.2
HI								
Week 2	<10 (0%)	<10 (0%)	28±13* (50%)	101±126* (83%)	25±53 (33%)	22±6* (50%)	121±63* # (100%)	26±13* (50%)
Week 4	18±2 (0%)	<10 (17%)	71±18* (83%)	71±102 (67%)	160±53* # (100%)	113±98* (100%)	640±436* # (100%)	139±144* (100%)
Week 8	80±22 (83%)	18±6 (33%)	202±87 (100%)	113±97 (83%)	254±34* (100%)	180±115 (100%)	557±443* (100%)	243±223* (100%)
Week 12	63±20 (83%)	20±0 (0%)	106±25 (100%)	113±41 (100%)	226±82* (100%)	80±47 (83%)	320±218* (100%)	279±88* # (100%)
VN								
Week 4	<40	<40	<40	<40	<40	<40	72±35* #	<40
Week 8	<40	<40	58±42	195±80*	228±40* #	47±49	134±19*	57±41

All mice were vaccinated once intramuscularly with dose of 0.5 µg HA H5N1 inactivated virus vaccine. Serum samples were collected from immunized mice and the antibody titers were determined by ELISA, HI and VN immunoassays. The data are presented as geometric mean titers (GMT) with standard errors (SE) of six mice per group. The seroprotection rate (SPR, %) is the percentage of mice achieving a post-vaccination titer ≥40.

*p<0.05: Comparison with the group without adjuvant at the same time point.

# *p*<0.05: Comparison with the group of alum at the same time point. <1,000 means undetectable in an initial dilution of 1∶1,000 on the ELISA assay system. <10 means undetectable in an initial dilution of 1∶10 on the HI assay system. <40 means undetectable in an initial dilution of 1∶40 on the VN assay system.

**Table 2** shows the HI antibody responses in mice immunized with different vaccine candidates formulated with or without adjuvant. 0.5 µg HA of non-adjuvanted inactivated virus elicited HI titers less than GMT of 20 within 4 weeks after administration. Afterwards, it increased to 80 at week 8, then, it decreased slightly to 63 at week 12. Similar to the findings of the IgG antibody titers, inactivated virus formulated with CpG could not enhance the HI titers, indicating that CpG has no adjuvanticity effect in 10-µg dosage. On the other hand, alum- or PELC(adsorption)- vaccines were capable of inducing higher HI titers than those obtained for virus alone, in particular in the early stage of the post-immunization (*p*<0.05); however, no significant difference was observed between alum- and PELC(adsorption)- vaccines. Significant difference (*p*<0.05) was found in the group of mice that received PELC/CpG(adsorption)- vaccines with respect to the group with alum. However, alum/CpG did not have this additional adjuvanticity effect. Also note that the potency of the PELC/CpG(encapsulation)- vaccines was rather reduced than PELC/CpG(adsorption)-ones, in agreement with the findings of IgG titers. In terms of the longevity of the induced antibody responses, there was no significant difference between response to the virus alone and versus the alum- or PELC-adsorbed formulation. In both cases, the HI titers peaked at Week 4 or Week 8 and fluctuated. Meanwhile, the antibody levels were induced slowly and then reached a high at Week 12 in the case of PELC-encapsulated formulation. Considering the seroprotection rate, only the mice received PELC/CpG(adsorption)- vaccines could easily and quickly reach 100%.

**Figure 3:**
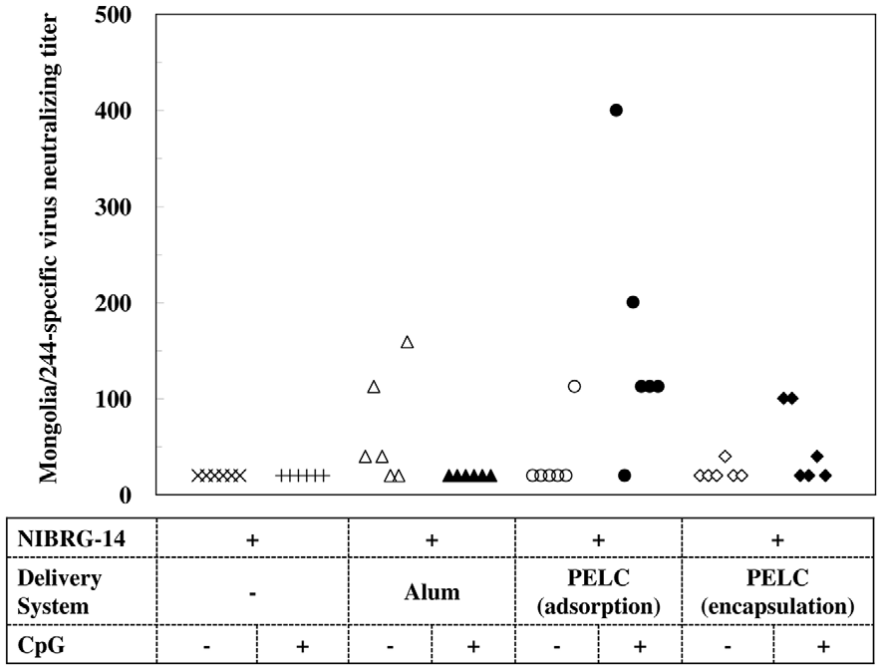
Mongolia/244-neutralizing antibodies elicited in mice following immunization with NIBRG-14 inactivated virus vaccine. BALB/c mice were vaccinated once i.m. with dose of 0.5 µg HA NIBRG-14 inactivated virus vaccine. At week 12, sera were collected from blood and incubated with a heterologous virus strain, Mongolia/244. An undetectable level was scored as a titer equal to 20.

As shown in **Table 2**, while alum or CpG alone does not enhance the VN titers, the addition of PELC (either adsorption or encapsulation) leads to much higher titers, in agreement with its adjuvanticity found in HI titers. Also, alum adsorbed with CpG could not increase the VN titers compared to alum alone. The highest VN responses were elicited in the group of PELC/CpG(adsorption) at Week 4 and in the group of PELC(encapsulation) at Week 8. In overall speaking, only PELC-formulated virus could induce sufficient and sustainable antigen-specific serological protective antibodies, adsorbed and encapsulated as well. In connexion with the cross-protective immunity, only the mice received PELC/CpG(adsorption)- vaccines could generate an effective cross-clade neutralizing antibodies (**Figure 3**), a feature similar to the findings of IgG2a/IgG1.

In summary, antigen-specific IgG, HI and VN antibody responses elicited in BALB/c mice following a single-dose H5N1 inactivated virus vaccine immunization have found that, first, CpG alone with 10 µg/dose has no adjuvanticity effect. Second, alum- or PELC-adsorbed inactivated virus could induce better antibody responses than those obtained from non-adjuvanted virus. Third, PELC-encapsulated inactivated virus could induce better antibody responses with respect to PELC-adsorbed vaccine. Fourth, PELC/CpG-adsorbed virus could induce better antibody responses than alum alone, whereas alum/CpG-adsorbed virus vaccine does not have this effect. Fifth, PELC/CpG-encapsulated inactivated virus could not induce better antibody responses with respect to PELC/CpG-adsorbed vaccine. Finally, only PELC/CpG-adsorbed vaccine allows the host to bias the IgG2a/IgG1 ratio and also allows to reach easily and quickly 100% of seroprotection against a homologous virus strain and effective cross-protection against a heterologous virus strain.

## Discussion

Following injection, vaccine antigens may directly act on the host immune cells such as antigen-presenting cells (APCs) or B cells, or undergo degradation [15]. However, the pathway taken may also be influenced by the presence of vaccine adjuvants, which trigger TLRs of the immune cells or act through other pathways such as antigen processing and presentation [9], [15]. Although the mechanisms of adjuvant action are often controversial, a common mechanism attributed to alum is the delivery/depot capability, in which mineral salts associate with antigen and effectively increase its biological and immunological “half-life” at the site of immunization [15]. Ideally, a single injection can achieve the prime-boost vaccination with the aid of a stepwise release of vaccine antigens. After alum, MF59 (Novartis, [9], [16]) and AS03 (GlaxoSmithKline, [17]) are two oil-in-water (O/W) emulsion-type adjuvants with significant potential in the development of pandemic influenza vaccines. O/W emulsions possess better efficiency than alum due to the induction of an early and strong immuno-competent environment at the site of injection by targeting muscle cells [16]. Studies have shown that MF59 helps elicit strong immune memory and sustained serological responses when used with seasonal and pre-pandemic influenza vaccines [9]. Clinical data have also demonstrated that pandemic H5N1 vaccines formulated with AS03 induce superior the rate of seroconversion and the titer of cross-neutralization antibody response than those obtained either with non-adjuvanted vaccines or alum-formulated vaccines [17]. The WHO Strategic Advisory Group of Experts on immunization recommended that use of O/W adjuvants was important in view of the anticipated limited vaccine availability at global level [18].

Besides targeting antigens to the immune system, emulsions are also capable of prolonging the duration of action of vaccine candidates [12], [15]. Freund's adjuvants and Montanide™ ISA 51 (ISA 51, developed from SEPPIC, France), water-in-oil (W/O) emulsions based on lipophilic mannide monooleate and mineral oil, have been evaluated to achieve long-term protective immune responses and to improve the innocuity of the vaccine [19]. ISA 51 is used in a non-small-cell lung cancer vaccine, CimaVax EGF, in Cuba and Chile [15]. TiterMax® is a squalene-based W/O emulsion stabilized by microparticulate silica and the nonionic block copolymer polyoxyethylene-polyoxypropylene-polyoxyethylene (POE-POP-POE, known as Pluronic® or Poloxamer®) [20]. However, no influenza vaccine formulated with this type of emulsions was developed since they are crowded in the oily phase and causing local reactions at the site of injection into animals. To improve the injectability of such vaccines, we have previously described the incorporation of hydrophilic polymeric emulsifier PEG-*b*-PLACL in the aqueous solution to alter the water affinity of W/O-adjuvanted vaccines, so that the pre-emulsified stock could be re-dispersed into aqueous before injection. The ameliorated W/O/W emulsion increases injectability and conceptually diminishes local reactions with respect to the W/O type vaccines produced from the same oil [12], [14].

Concerning the role of adjuvants in vaccine immunogenicity, non-adjuvanted inactivated H5N1 whole virus vaccines have showed the priming responses of dose-dependent IgG titers with doses ranging from 0.5 to 5 µg HA, but VN antibodies were below the detection limitation. The data are similar to a study where the prime/boost of Vero-cell derived H5N1 vaccines with doses ranging from 0.001 to 3.75 µg HA antigens without adjuvant [4]. The IgG antibodies then increased strongly and VN antibodies were also detected after the boost dose. Nonetheless, no boost effect was detected in the cases of low-dose (0.5 µg HA antigen) alum- and PELC-adjuvanted vaccines. Meanwhile, high-dose (5 µg HA antigen) PELC-adjuvanted vaccine could overcome this situation to show the boost effect. The IgG and VN data showed a clear dose-response for a single injection of PELC-formulated inactivated virus vaccines of 0.5 µg HA and 5 µg HA in mice, and also after the boost dose; however, it is not the case for alum-adjuvanted vaccines since no significant differences (*p*≈1) in IgG, HI and VN titers between the two doses. Regarding the adjuvant combination systems, i.e. additional component CpG was added to the delivery systems such as alum and PELC, the humoral response data implied that 10 µg of CpG alone has no adjuvanticity effect with inactivated NIBRG-14 vaccine candidate (**Table 2**). We also found that alum-adsorbed CpG did not enhance the antibody responses with respect to alum alone, whereas PELC-adsorbed CpG induced significant immune responses (antigen-specific antibody titers, IgG2a/IgG1 ratio, and cross-clade neutralizing titers when compared to no adjuvant or to alum (*p*<0.05); however, it is not the case when CpG was co-encapsulated into PELC emulsion. It is beneficial of just adding the intracellular receptor TLR9 agonist CpG to the ready-to-use PELC emulsion as such. We have also attempted to determine whether T-cell responses could also be manipulated when antigen was formulated with designed adjuvant systems. Our findings indicate that even antigen-specific T-cell proliferative responses were significantly enhanced after immunization with PELC(encapsulation)- or PELC/CpG(adsorption)- adjuvanted inactivated virus; however, the IFN-γ concentration in the splenocyte supernatants was measured at the same level as virus alone (data not shown). It still needs to be evaluated whether the encapsulation of influenza virion and/or CpG is beneficial on the host immune cells such as APCs or T cells when compared to adsorption of the same antigen and/or immunostimulator.

We conceive the preparation of a W/O/W emulsion requires two factors: a two-step preparation process and an emulsifying system with an intermediate HLB (hyhrophilic-lipophilic balabce) value, as summarized in **Table 3**. The two-step preparation process comprises a homogenizing (or called emulsifying) step and a diluting (or called dispersing) step. This procedure offers the opportunity to individually identify each parameter of the process when used for the research and development of new formulations. This implies that the Novartis MF59® adjuvant, which contains 4.3% squalene, 0.5% hydrophilic emulsifier Tween® 80 (polyoxyethylene sorbitan monooleate, HLB_Tween®80_ = 15) and 0.5% lipophilic emulsifier Span®85 (HLB_Span®85_ = 1.8), renders stable O/W submicron emulsion via a single-step manufacture process [9], [21]. Thereafter, we found an emulsifying system of intermediate HLB value is also required to achieve the W/O/W multi-phase emulsion. For example, the GSK ASO3 adjuvant is a fluid O/W emulsion, in which a pre-emulsified stock comprising squalene, α-tocopherol, and Tween®80 was mixed with a bulk antigen before injection [17]. This adjuvant is prepared via a two-step manufacture process; however, it lacks an emulsifying system with an intermediate HLB value (both squalene and α-tocopherol act as core oil). Consequently, the development of PELC allows of optimization of a process for making a multi-phase W/O/W emulsion, which may trap and/or encapsulate antigens and/or bioactive substances in the multi-phase emulsion. One should note the canonical two-stage emulsification procedure which described in the literature consists of two individual stirring emulsification processes. Firstly, the W/O emulsion is prepared by emulsifying an *internal aqueous solution* and a low-HLB surfactant in oil. Secondly, the primary W/O emulsion is re-emulsified in an *external aqueous solution* containing a high-HLB surfactant to produce a W/O/W multiple emulsion. The stirring process is usually carried out in a high-shear device to produce fine and stable droplet, which might damage any bioactive substance to be encapsulated or adsorbed. Here the immunological evaluation results demonstrated that encapsulating inactivated H5N1 influenza virus and/or CpG into these emulsified nanoparticles critically influences the host immune responses. To extend this aspect to the temporary therapeutic applications in sustained delivery, the aqueous solution may be an aqueous medium alone, such as PBS, or an aqueous medium containing an antigen or a bioactive substance such as peptides, anticancer agents, hormones, or other active agents such as antibiotics or antiparasitics. The antigen or the bioactive substance may be incorporated into the multiphase emulsion via dissolving in either the oily or the aqueous phase. Last but not least, it is also worth noting that the antigens are exposed low shear stress (6,000 rpm) during emulsification processing of PELC emulsion stock.

**Table 3:** Comparison of components, dispersion type, and manufacture process of selected examples of emulsions.

Adjuvant	Company	Components			Dispersion type	Manufacture process
		Core oil	Hydrophilic Emulsifier	Lipophilic Emulsifier		
ISA51	SEPPIC	Drakeol	-	Mannide monooleate	W/O	Single-step emulsification process
MF59	Novartis	Squalene	Tween®80	Span®85	O/W	Single-step emulsification process
AS03	GlaxoSmithKline	Squalene/α-tocopherol	Tween®80	-	O/W	Emulsification-dispersion process
PELC	VRDC, NHRI	Squalene	PEG-b-PLACL	Span®85	W/O/W	Emulsification-dispersion process

So far as the safety issue is concerned, a claim was made that adjuvanted vaccines foster either accepted or hypersensitive autoimmune diseases due to boost the immune system of the body too much [3]. Of note, clinical studies also found that vaccines containing CpG arise in a severe autoimmune disease, Wegener's granulomatosis, in which blood vessels become inflamed [3]. Thus, encapsulating small amount of such immunostimulatory adjuvant into biological inert vehicle of PELC is a probable strategy to improve the innocuity of vaccines. From a viewpoint of emulsion composition, the core oil selected, squalene, is a naturally occurring oil produced by plants and is also produced abundantly by human beings, notably the precursor of cholesterol and steroid hormones synthesized in the liver and the skin [22]. Although some studies indicated that anti-squalene antibodies were detectable in the sera of individuals with the so-called Gulf War syndrome, anti-squalene antibodies are not increased by immunization with vaccines with the squalene-in-water emulsion was also reported [22]. The excipient use of Span®85 in the oily phase is also positively indicated, as it is an emulsification agent widely used in pharmaceutical formulations and also in licensed human vaccines [9], [21]. Eventually, biodegradable PEG-*b*-PLACL is derived from FDA-approved PEG, polylactides, and poly(ε-caprolactone) [12], [14]. It allows stabilization of emulsion particles during storage but allows disintegration of the system post-injection [13]. Consequently, PELC-formulated vaccines comprising the highly safe components manifested its potential safety and efficacy.

The data presented here demonstrate that the antigen-specific serum antibody responses elicited after two doses of non-adjuvanted vaccine were lower than those observed after a single dose of adjuvanted vaccine, PELC and alum as well. Moreover, encapsulation of inactivated H5N1 influenza virus and CpG into emulsified nanoparticles critically influences the antigen-specific and cross-clade serological antibody responses against pandemic influenza; the use of PELC could be more adaptable in preparation for a potential shortage of prophylactic vaccines against local infectious diseases, in particular pandemic influenza. Further investigations are under way to examine the micro-encapsulation technology for a single-dose multivalent vaccine against influenza and local infectious diseases where they co-exist.

## Materials and Methods

### Vaccine preparation

The vaccine used in this study was a formalin-inactivated whole virus vaccine, NIBRG-14 (kindly supplied by the UK National Institute of Biological Standard and Control, NIBSC), derived from a reassortant H5N1 vaccine strain containing modified HA and neuraminidase (NA) from the highly pathogenic avian influenza strain A/Vietnam/1194/2004 virus. The NIBRG-14 vaccine viruses were grown in eggs by the NIBSC and supplied to Taiwan Centers for Disease Control (CDC). Taiwan CDC further amplified the NIBRG-14 for four generations in eggs and then transferred to Vaccine Research and Development Center (VRDC) of National Health Research Institutes (NHRI). In VRDC, the received NIBRG-14 was propagated in serum-free media (Cesco, Taiwan) and in the MDCK cell culture-based roller bottle technology. The manufacturing space is specified as P2+ facility with one positive pressure cell culture room, two negative pressure rooms for virus growth and downstream purification processes, two air-lock rooms, and two buffer rooms. The VRDC's MDCK cells were purchased from Food Industry Research and Development Institute (FIRDI), Taiwan. Master and working cell banks were established following current Good Manufacturing Practices (cGMP) guidelines. Formalin-inactivated vaccines were prepared with 0.1% formalin at 37°C for 24 hr. We had performed plaque assay based on plaque forming unit (PFU) in MDCK cells and the TCID_50_ (50% tissue culture infective dose) assay based on the cytopathic effect to evaluate whether there was incomplete formalin inactivation during storage. After the sterilization through a 0.22 µm filter membrane, the HA content of the vaccine bulk was determined by single-radial diffusion (SRD) assay with the standard antigen and antiserum from NIBSC. The antigen medium was prepared with particular HA concentration of the vaccine bulk which is diluted in the phosphate buffered saline (PBS).

### Adjuvant preparation

Murine CpG ODN was synthesized by Invitrogen Taiwan Ltd and given as a 10 µg per dose dissolved in the PBS or in the antigenic media. The CpG ODN sequence used was 5′-TCC ATG ACG TTC CTG ACG TT-3′ with all phosphorothioate backbones. Alum (aluminum phosphate) suspension was kindly provided from Taiwan CDC and given as a 300 µg per dose in the acidic media (pH = 6).

PELC is a squalene W/O/W nanoemulsion stabilized by Span®85 (sorbitan trioleate, Sigma-Aldrich, Steinheim, Germany) and PEG-*b*-PLACL, the latter consisting 75 wt-% of hydrophilic bioabsorbable PEG and 25 wt-% of lipophilic biodegradable PLACL with molecular weight of 7,000 daltons as previously described [12]–[14]. Briefly, 120 mg of PEG-*b*-PLACL, 0.8 mL of aqueous solution (*internal aqueous solution*), and 1.1 mL of oily solution consisting of squalene (Sigma-Aldrich, Steinheim, Germany) and Span®85 (85/15 v/v) were emulsified using Polytron®PT 3100 homogeniser (Kinematica AG, Switzerland) under 6,000 rpm for 5 min. The emulsified PELC formulation was stored at 4°C until use. PELC-formulated vaccine was investigated by re-dispersing 200 µL of stock emulsion into 1800 µL of aqueous solution (*external aqueous solution*) and mixed with a test-tube rotator (Labinco LD-79, Netherlands) under 5 rpm at least 1 hr before injection. Inactivated influenza virus and/or CpG were introduced either in the *internal aqueous solution* or the *external aqueous solution* to yield a PELC-encapsulated or adsorbed influenza vaccine, respectively. The size distribution of the emulsions was investigated by microscopic aspects (Olympus DP70 Digital Microscope Camera, Melville, NY, USA) and the laser light scattering technique using a Brookhaven 90 plus particle size analyzer (Brookhaven Instruments Limited, New York, USA).

### Ethics statement and immunizations

All experiments were conducted in accordance with the guidelines of Laboratory Animal Center of NHRI. The animal use protocols have been reviewed and approved by the NHRI Institutional Animal Care and Use Committee (IACUC). Five weeks old female BALB/c mice were obtained from the National Laboratory Animal Breeding and Research Center (Taipei, Taiwan) and acclimatized for at least one week at the NHRI animal facility prior to use. To investigate the potency of candidate H5N1 influenza vaccine, we designed two immunological experiments. In the first experiment, mice were primed intramuscularly (i.m.) with one of two different doses (0.5 µg or 5 µg HA), either with antigen in PBS or adsorbed with alum or PELC. At week 30, all mice were boosted i.m. with dose of 0.5 µg HA H5N1 non-adjuvanted inactivated virus vaccine. In the second experiment, all mice were vaccinated i.m. once with 0.5 µg HA given with or without adjuvant. Serum samples were collected from immunized mice and the antibody titers were determined by enzyme-linked immunosorbent assay (ELISA), HI titration, and VN assays.

### ELISA immunoassay

The presence of NIBRG-14-specific antibodies in the sera was determined by ELISA. In brief, 100 µL of dilute inactivated virus (1 µg/mL) were coated in 96-well microtiter plates with 0.05 M carbonate buffer (pH 9.6, Sigma, St. Louis, MO, USA) by overnight incubation at room temperature. Coated plates were washed once with PBS containing 0.05% Tween®20 (Sigma, St. Louis, MO, USA) and then blocked with 1% bovine serum albumin (BSA, Sigma, St. Louis, MO, USA) in PBS at room temperature for 2 h. Diluted sera (starting dilution 1∶1000, serial two-fold serum dilutions) from immunized animals were applied to wells at room temperature for 2 h. Followed by HRP-conjugated goat anti-mouse IgG (ICN Cappel, Aurora, Ohio, USA, 1∶5,000), the assay was developed with substrate solution 2,2′-azino-di(3-ethyl-benzthiazoline-6-sulfonate (ABTS® Peroxidase, KPL, MD, USA) for 20 min at room temperature (avoid light). Plates were read at 405 nm using an ELISA plate reader (Thermo Multiskan® spectrophotometer, Vantaa, Finland). For IgG subclass determination, 100 µL of HRP-rabbit anti-mouse IgG1 (AbD Serotec, Kidlington, UK, 1∶5,000) or HRP-rabbit anti-mouse IgG2a (AbD Serotec, Kidlington, UK, 1∶2,000) was added. The titers were determined from the reciprocal of the final dilution that gave an optical of two-fold absorbance of pre-immune sera. For calculation purposes, an undetectable level was scored as a titer equal to 500.

### HI titration

The principle of the HI test is based on the ability of specific anti-influenza antibodies to inhibit hemagglutination of turkey red blood cells (RBCs) by influenza virus HA. Non-specific inhibitors of agglutination were removed by heat treatment and addition of receptor-destroying enzyme. After pretreatment, serum samples (two-fold dilutions starting with an initial dilution of 1∶10) were incubated with four HA units of influenza strain. Turkey RBCs were then added and the inhibition of agglutination was scored. The serum titer was expressed as the reciprocal of the highest dilution that showed complete inhibition of HA. For calculation purposes, an undetectable level was scored as a titer equal to five. The seroprotection rate (SPR, %) was calculated from the proportion of mice achieving a post-vaccination titer ≥40.

### VN assay

The 200 TCID_50_ per well of NIBRG-14 virus were incubated with two-fold-diluted mice sera at a starting dilution of 1∶40. Mixtures of virus and serum were transferred to monolayers of MDCK cells and incubated at 37°C and 5% CO_2_ for 4 days. The neutralizing titer was defined as the reciprocal of the highest serum dilution at which the infectivity of the H5N1 virus' 200 TCID_50_ for MDCK cells was completely neutralized in 50% of the wells. Infectivity was identified by the presence of cytopathy on Day 4 and the titer was calculated using the Reed-Muench method. For calculation purposes, an undetectable level was scored as a titer equal to 20. To assess cross-protective immunogenicity, serum samples were also tested with a heterologous Mongolia/244 (ST/NIAID, St Jude Children's Research Hospital, National Institute of Allergic and Infectious Disease, USA) derived by reverse genetics from a drifted H5N1 clade 2.2 isolate A/Whooper swan/Mongolia/244/2005 and H1N1 A/Puerto Rico/8/34.

### Statistical analysis

Statistical significance (*p*<0.05) was determined by performing two-tailed Student's *t*-test on log-transformed values, using Microsoft Excel.
